# Interproximal biofilm removal by intervallic use of a sonic toothbrush compared to an oral irrigation system

**DOI:** 10.1186/s12903-015-0079-6

**Published:** 2015-08-05

**Authors:** Pune N Tawakoli, Bärbel Sauer, Klaus Becker, Wolfgang Buchalla, Thomas Attin

**Affiliations:** Clinic of Preventive Dentistry, Periodontology and Cariology, University of Zurich, Center of Dental Medicine, 8032 Zurich, Switzerland; Department for Conservative Dentistry and Periodontology, University Medical Center, University of Regensburg, Regensburg, Germany

**Keywords:** alamarBlue assay, Intervallic biofilm removal, Interproximal biofilm, Oral irrigation system, Sonic toothbrush

## Abstract

**Background:**

The purpose of this in-vitro study was to investigate the potential of biofilm removal in interproximal tooth regions using intervallic cleaning with an oral irrigator or a sonic toothbrush.

**Methods:**

Three-species biofilms (*Streptococcus mutans* (OMZ 918), *Streptococcus oralis* SK 248 (OMZ 60), *Actinomyces naeslundii* (OMZ 745)) were grown on hydroxyapatite discs for 3 days in culture media. Every 24 h, specimens were incubated for 15 min in resazurin solution (i.e., culture medium and 10 % v/v alamarBlue®) to measure the metabolic activity with a fluorescence spectrophotometer in relative fluorescence units (rfu) at baseline. Then, specimens were fixed in interproximal holding devices and underwent treatment with an oral irrigator (WF; Waterpik® Sensonic WP-100E), an active sonic toothbrush (WPa), or an inactive sonic toothbrush (WPi; Waterpik® Sensonic SR-3000E) for 10 s (*n* = 18/group). Untreated biofilms served as controls (CO). After treatment, bacterial activity was re-measured, and specimens were re-grown in fresh medium for 24 h until next cleaning procedure. Altogether, cleaning was repeated in intervals of three treatment days (d1, d2, d3). After d3, SEM images were taken (*n* = 8) and CFU was measured (*n* = 3). Metabolic activity was analyzed for each disc separately, rfu values were averaged for d1 to compare initial biofilm stability, and ratios of baseline and post-treatment values were compared. Results were analyzed using ANOVA with the post-hoc Scheffé test, or Kruskal-Wallis with post-hoc Mann–Whitney test.

**Results:**

Median baseline rfu-values of d1 resulted in 7821.8 rfu (interquartile range = 5114.5). Highest reduction in metabolic activity was recorded significantly for the oral irrigator used for 10 s (residual activity per day d1: WF 17.9 %, WPa 58.8 %, WPi 82.5 %, CO 89.6 %; d2: WF 36.8 %, WPa 85.2 %, WPi 82.5 %, CO 90.0 %; d3: WF 17.2.%, WPa 79.6 %, WPi 96.3 %, CO 116.3 %). SEM images of untreated specimens (CO) and specimens treated with the sonic toothbrush (WPa and WPi) showed huge amounts of biofilm, while oral irrigator-treated specimens (WF) revealed barely any bacteria. CFU data confirmed the graduations between the groups.

**Conclusions:**

Cleaning of interproximal regions achieved better success with an oral irrigator as compared to the use of a sonic toothbrush. (350/ 350 words)

**Electronic supplementary material:**

The online version of this article (doi:10.1186/s12903-015-0079-6) contains supplementary material, which is available to authorized users.

## Background

Caries and periodontitis are caused by bacterial biofilms, accumulating on tooth surfaces and oral soft tissues. Since most oral hygiene devices do not sufficiently reach all niches and angles in the oral cavity mechanically, interproximal regions are often only affected by the properties of toothpaste slurry and the hydrodynamic forces produced during tooth brushing. To determine the highest hydrodynamic effects, many studies investigated the effect of sonic and manual tooth brushing on biofilms as well as differences within various types of sonic toothbrushes. Depending on the sonic toothbrushes’ type, side-to-side toothbrushes result more often in higher biofilm reduction than 50 %, while multidimensional toothbrushes remove less biofilm [1–3]. Comparing different side-to-side sonic toothbrushes among each other shows significant differences between the models ranging from 9 to 80 % [4]. However, until now, most investigations on the so called ‘non-contact biofilm removal’ were performed not using interproximal devices, but e.g. sonic toothbrushes, installed with defined distances directly adjusted towards the center of the biofilm coated disc surface [3–7]. Using this approach, the hydrodynamic forces, formed by the oral devices have direct access to the biofilm surface and strike, depending on the distance, with full intensity. Although describing a non-contact brushing approach, it still might differ from actual interproximal situations. Adams et. al [1] investigated the effect of monospecies-biofilm removal using an interproximal model with various distances from the bristle tips. Analyzing the emerging bubble velocities of the different sonic toothbrushes, they estimated shear stress values between 0.5 and 0.9 Pa, resulting in biofilm reduction up to 57 % for side-to-side and 16 % for oscillating-rotating toothbrushes in a distance of 0 - 5 mm. Frey (2012) developed an interproximal tooth device with an integrated shear stress sensor and analyzed shear stress in interproximal distances of 0.2 mm using different side-to-side toothbrushes [8]. Depending on the type of toothbrush and its mode of action, shear stress values of up to 10 Pa were measured. However, more pronounced shear stress values with higher biofilm removal might be assessed by higher fluid flow produced by oral irrigation systems (ORS). Studies investigating the use of dental water jets indicated reduced pro-inflammatory mediators, such as IL-1ß and PGE_2_ and removal of salivary plaque biofilm over 99 % independently of the water jet tip used [9–11]. While ORS were mainly analyzed in clinical trials, determining the outcome on reduction of bleeding, gingivitis and plaque biofilm, interproximal biofilm removal was not investigated yet [12, 13].

Therefore, the aim of this in-vitro study was to investigate the effect of an oral irrigator and a side-to-side toothbrush on multispecies biofilm removal using an interproximal tooth device. Moreover, treatment cycles using the ORS or sonic toothbrush were repeated in intervals of 24 h to simulate and analyze the effect of repeating oral hygiene patterns.

## Methods

### Biofilm formation

Bacterial strains were obtained from the Institute for Oral Biology, Section for Oral Microbiology and General Immunology, University of Zürich, Zürich, Switzerland. Before biofilm formation, the strains (*Streptococcus mutans* OMZ 918, *Streptococcus oralis* OMZ 607, *Actinomyces naeslundii* OMZ 745) were gained from precultures streaked on Columbia sheep’s blood agar (CSBA) plates (bioMérieux, Marcy l’Etoile, France). Colonies were propagated planktonic in a substrate composed of 30 % saliva solution and 70 % modified fluid universal medium (mFUM) [14] separately on a rocker at 37 °C in jars using gas-paks to create anaerobic conditions (GENbox anaer and GENbag anaer, bioMérieux, Marcy l’Etoile, France). Therefore, fresh saliva was gained by one healthy donor and centrifuged two times for 30 min by 13400 rpm. Following the opinion of the Ethics Committee of the Canton of Zurich, Switzerland, no ethical approval is needed for the donation of saliva as explained above (no. 0324/2013 and no. 50/14). The pellet was removed each time and the remaining supernatant was diluted 1:2 in sodium chloride (0.9 % NaCl) prior to sterile filtration (TPP syrenge filters with 0.2 μm pores, Faust, Schaffhausen, Switzerland). The resulting saliva solution was used in all experimentations. FUM, a well-established tryptone-yeast based broth medium was described by Loesche et al. [15]. FUM contained (per liter of distilled water): 10 g of tryptone, 5 g of yeast extract, 3 g of glucose, 2 mg of hemin, 1 mg of menadione, 0.5 g of cysteine hydrochloride, 0.1 g of dithiothreitol, 2.9 g of 0.9 % NaCl, 0.5 g of Na_2_CO_3_, 1 g of KNO_3_, 0.45 g of K_2_HPO_4_, 0.45 g of KH_2_PO_4_, 0.9 g of (NH_4_)_2_SO_4_, and 0.188 g of MgSO_4_ * 7H20. It was modified by supplementing 67 mmol/l Sørensen’s buffer to a final pH of 7.2. Glucose was replaced by 3 g of a 1:1 mixture of glucose and sucrose. The modification used in this study was adopted from the Zurich biofilm protocols [14]. After approximately 6 - 7 h the bacterial solutions were adjusted to the optical density (OD550) of 1 and mixed in a tube as inoculum. To quantify the inocula per ml, colony forming units (CFU) were plated out on CSBA plates and incubated anaerobically in jars using gas-paks (t = 2 d). In the meantime, sterile sintered hydroxyapatite discs (Ø 5 mm, Clarkson Chromatography Products, South Williamsport, USA) were incubated in 800 μl of non-stimulated saliva solution for 4 h at gentle agitation to form a pellicle (100 rpm at room temperature). For biofilm formation, pellicle-coated discs were then placed in new 24-well polystyrene cell culture plates and incubated with 1 ml of the prepared inocula during gentle agitation for 24 h in jars at 37 °C using gas-paks (GENbox anaer and GENbags anaer, bioMérieux, Marcy l’Etoile, France). Media was refreshed daily prior to treatment procedures and directly after treatment by transferring the specimens in new plates filled with fresh media (30 % saliva solution + 70 % mFUM). pH was controlled daily in the overnight medium directly after the first media change using a pH meter (Mettler-Toledo Easy Five, Mettler-Toledo AG, Schwerzenbach, Switzerland).

### Treatment

**Fig. 1:**
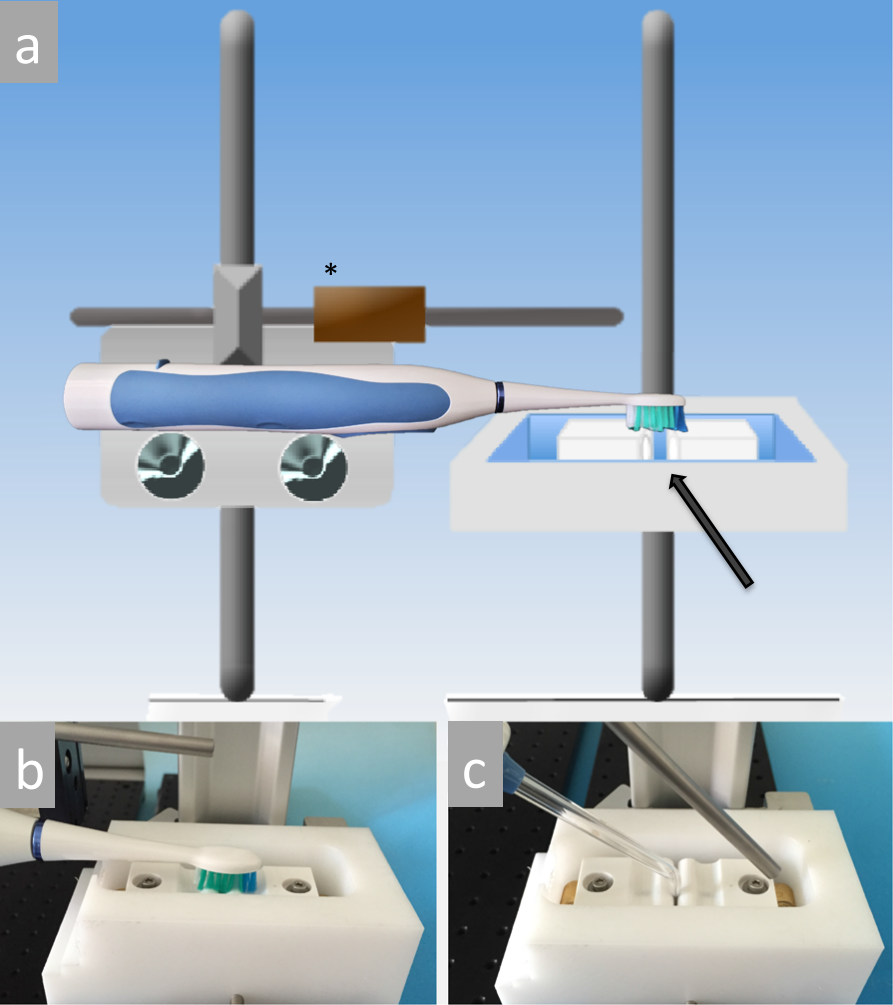
**a** Draft of the used holding device with an adjustable load (*); Interproximal specimen position within the chamber (arrow). Oral devices can be positioned perpendicularly to the fixated specimens, as illustrated in **b**) for the sonic toothbrush and **c**) for the oral irrigator

Specimens were divided into four groups. Three independent experiments were performed to obtain *n* = 18 specimens per group (first experiment *n* = 4, second *n* = 6, last experiment *n* = 8 specimens per group). Each experiment consisted of three treatment days (d1, d2, d3). Prior to each treatment, measurements of the metabolic activity were performed to obtain baseline values for each specimen. Then, specimens were placed carefully into an interproximal device with 2 specimens in a distance of 0.5 mm face to face (Fig. 1). The brushing device for electric toothbrushes was build in a co-operation between the Institute of Fluid Dynamics, ETH Zürich and the Department of Preventive Dentistry, Periodontology and Cariology of the University of Zurich, Switzerland. For experimentation, 25 ml water of 36 °C was pipetted into the device to cover the interproximal regions and the specimens. For the WF-group, the oral irrigator (Waterfloss, Waterpik® Sensonic WP-100E) was adjusted using the JT-100E Classic Jet Tip at a 90° angle towards the interproximal region as described in the manufacturer’s information. The pressure control was positioned at level 10 (highest water pressure) and activated for 10 s. Afterwards, the specimens were carefully taken from the interproximal device and restored in plates with 0.9 % NaCl. For the WPa-group, the sonic toothbrush (Waterpik® Sensonic SR-3000E) was adjusted onto the device using the respective standard brush head with a load of the brush head onto the interproximal region of < 0.9 N as measured for sonic toothbrushes (total load 70 ± 5 g) [2, 16]. The brushing was performed for 10 s under static conditions. For the specimens of the WPi-group, the procedures were repeated for the inactivated brushes (power off). Specimens without treatment were used as control group (CO).

### alamarBlue assay and colony forming units

Prior to experimentation, the alamarBlue assay was validated in preliminary tests [see Additional file 1]. The above-described inoculum was diluted 1:2 in phosphate buffered saline in seven series. Samples of each dilution series were determined by colony forming units, resulting in dilution series from 5.5 to 350 × 10^6^ CFU/ml. Ten samples of each series were then used for kinetic measurements. Therefore, samples were incubated in wells containing 10 vol.% alamarBlue Cell Viability Assay Reagent (Life technologies, Zug, Switzerland) and measured in a Spectrophotometer with plate-reader at 560 nm excitation/585 nm emission at 37 °C (Spectramax M2, Molecular Devices, Bucher Biotec, Basel, Switzerland). Measurements were conducted every 15 min for 2 h. The measured relative fluorescence units of each dilution were plotted against the incubation time. The findings of these preliminary tests showed constant initial rates of the enzymatic reaction within a range of approximately 30 % of the total substrate conversion for each dilution curve [see Additional file 1]. To gain measurements within the range of linear increase for the current experimentations, the incubation time in the alamarBlue solution was set to 15 min.

For experimentation, biofilm coated hydroxyapatite discs were first stored in a new well plate with 0.9 % NaCl to avoid further growing during treatment. Then, specimens were transferred carefully into 96-well plates and incubated in 300 μl alamarBlue solution containing fresh media (30 % saliva solution + 70 % mFUM) with 10 vol.% alamarBlue under anaerobic conditions. Additionally, two wells were filled with blank alamarBlue solution (without specimens) and one well was filled with centrifuged bacteria from the planktonic inocula in alamarBlue solution to gain values for the maximal metabolic activity. After 15 min, 200 μl of each alamarBlue solution was pipetted into new 96-well plates and metabolic activity was measured in a Spectrophotometer with plate-reader at 560 nm excitation/585 nm emission. The resulting relative fluorescence units (rfu) were defined as baseline values or pre-treatment rfu. After measurements, specimens were stored in 0.9 % NaCl and further experiments were conducted (treatments using the different oral devices). Then, post-treatment values were obtained by using the alamarBlue assay as described before. After rfu-measurements, specimens were transferred to fresh substrate to enable regrowth and retreated with the identical procedures 24 h later. Therefore, specimens were distributed into the same groups. Altogether, each specimen underwent three treatment cycles (d1, d2, d3).

After the last treatment and measurement using alamarBlue (d3), three specimens of each group were analyzed additionally using CFU. Therefore, specimens were vortexed in 1 ml 0.9 % NaCl for 2 min and sonified for 5 s. Each bacterial suspension was diluted in 0.9 % NaCl and plated out on Columbia sheep’s blood agar (CSBA) plates (bioMérieux, Marcy l’Etoile, France). CSBA plates were then incubated in jars at 37 °C, using gas -paks (GENbag anaer, bioMeriux, Marcy l’Etoile, France) for 2 days.

### Scanning electron microscopic analysis

For SEM, two samples per group (*n* = 8) were used after the last treatment cycle (d3). Specimens were washed with 0.9 % NaCl solution and fixed in 4 % glutaraldehyde solution (in 0.1 M sodium potassium phosphate buffer, pH 7.0) for at least 24 h. Dehydration was achieved gradually (2 × 15 min in ethanol 50 vol.%, 2 × 15 min in ethanol 70 vol.%, 2 × 15 min in ethanol 80 vol.%, 2 × 15 min in ethanol 90 %, 3 × 20 min in ethanol 96 % and 2 × 60 min in ethanol absolute). Prior to gold sputter coating the critical point drying was performed. Specimens of all groups were examined after the last periodically repeated treatment step (d3). Additionally images of specimens without bacteria were taken. Magnifications of 45x were taken to image the specimen surfaces and 500x to show more detailed surface characteristics.

### Statistical analysis

Metabolic activity was analyzed for each disc separately, and the ratio of post-treatment to baseline values were compared within the treatment days (d1, d2, d3). Analysis of the data was performed using ANOVA with the post-hoc Scheffe test, or Kruskal-Wallis with post-hoc Mann–Whitney test.

## Results

**Fig. 2:**
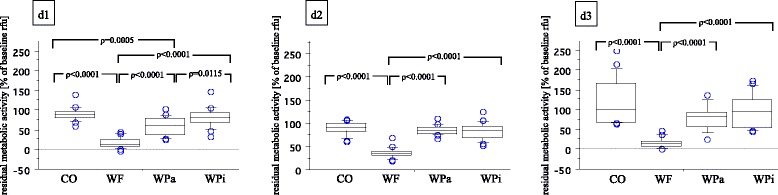
Boxplots of residual metabolic activity in % of baseline rfu-values of different experimental conditions with median (inner horizontal line), 1^st^ and 3^rd^ quartile (lower and upper box line) and 10^th^ and 90^th^ percentile (whiskers). WF = oral irrigator; WPa = sonic toothbrush, active; WPi = sonic toothbrush, inactive; CO = Control. Significant differences are illustrated above corresponding boxplots

**Fig. 3:**
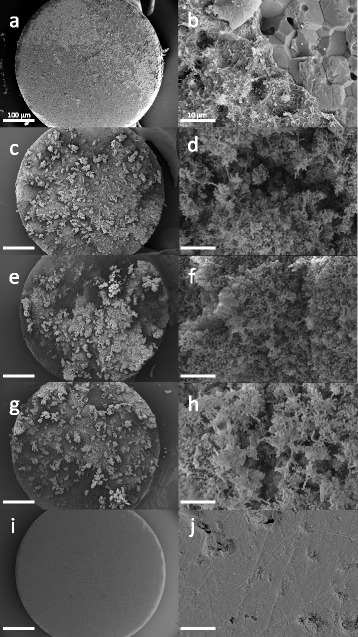
SEM images of all groups after the last treatment (d3). Specimens after treatment with the oral irrigator WF (**a**,**b**), the active sonic toothbrush WPa (**c**,**d**), the inactive sonic toothbrush WPi (**e**,**f**), the control group CO without treatment (**g**,**h**) and specimens without bacteria (**i**,**j**). Areas with shorn-off biofilm are shown in **a**) and **b**). Magnifications of 45x (a,c,g,e,I; scale bar = 100 μm) and 500x (b,d,f,h,j; scale bar = 10 μm) were used

**Fig. 4:**
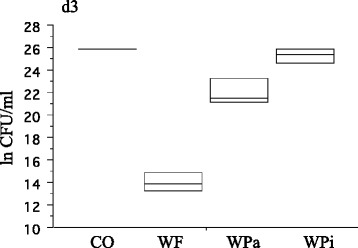
Boxplots of residual bacteria after d3 in ln CFU/ml (*n* = 3 per group). Two specimens of the CO-group revealed uncountable plates (>10^11). The median CFU (inner horizontal line) resulted in 1.0 × 10^6 (WF), 2.2 × 10^9 (WPa), 1.1 × 10^11 (WPi) and 1.8 × 10^11 (CO). WF = oral irrigator; WPa = sonic toothbrush, active; WPi = sonic toothbrush, inactive; CO = Control

Median baseline rfu-values of d1 (all groups) resulted in 7821.8 rfu (interquartile range = 5114.5). Baseline rfu-values of d2 (pre-treatment rfu) showed higher metabolic activity than d1, irrespective of the treatment group (mean ± SD, WF: 12045 ± 4414, WPa: 11832 ± 4331, WPi: 10600 ± 3362, CO: 10508 ± 3153). Baseline rfu-values of d3 revealed reduced metabolic activity compared to d1 and d2 (mean ± SD, WF: 5864 ± 3974, WPa: 6768 ± 3753, WPi: 6531 ± 4490, CO: 6878 ± 4093; Table 1). Post-treatment rfu-values were related to baseline rfu-values to calculate the residual metabolic activity in percentage. Significantly highest reduction in metabolic activity with regard to baseline was shown for the WF-group (oral irrigator) for 10 s for all treatment cycles (d1: 17.9 %, d2: 36.8 % and d3: 17.2 %). The WPa-group (active sonic toothbrush) showed significantly reduced metabolic activity on d1, whereas no significant reduction was measured on treatment cycle d2 and d3 (d1: 58.8 %, d2: 85.2 %, d3: 79.6 %). Specimens treated with the inactive sonic toothbrush (Wpi) and untreated specimens (CO) showed no significant reduction in biofilm activity at all (d1: WPi 82.5 %, CO 89.6 %; d2: WPi 82.5 %, CO 90.0 %; d3: WPi 96.3 %, CO 116.3 %; Fig. 2). Scanning electron microscopic images of the WF-group revealed almost biofilm-free surfaces with residual bacteria and partially shorn-off matrix on the outer areas (Fig. 3 a and b). Images of the WPa-, WPi- and CO-group showed huge amounts of biofilm with peaks of bacterial islands and aggregates (Fig. 3). Median CFU data resulted in 1.0 × 10^6 (WF), 2.2 × 10^9 (WPa), 1.1 × 10^11 (WPi) and 1.8 × 10^11 (CO). Two specimens of the CO-group were uncountable (>10^11; Fig. 4).

**Table 1:** Mean ± SD of pre- and post-treatment in relative fluorescence units [rfu] for the different devices at time points d1-d3

Treatment days	Groups	Pre–treatment [rfu] mean ± SD		Post-treatment [rfu] mean ± SD	
d1	WF	7077 ±	2564	1498 ±	1484
	WPa	7384 ±	2434	4715 ±	2841
	WPi	6832 ±	3202	5944 ±	3244
	CO	6669 ±	3157	5930 ±	2845
d2	WF	12045 ±	4414	4540 ±	2451
	WPa	11832 ±	4331	9966 ±	3414
	WPi	10600 ±	3362	8953 ±	3788
	CO	10508 ±	3153	9609±	3564
d3	WF	5864 ±	3974	885 ±	564
	WPa	6768 ±	3753	4627 ±	2087
	WPi	6531 ±	4490	4757 ±	1832
	CO	6878 ±	4093	5979 ±	2318

*WF* oral irrigator, *WPa* sonic toothbrush active, *WPi* sonic toothbrush inactive, *CO* Control

## Discussion

Lowest residual metabolic activity of interproximal biofilms on d1, d2 and d3 was achieved using the oral irrigator. Significant reduction in activity was also shown after treatments on d1 with the active sonic toothbrush. CFU data of the specimens after treatment on d3 mirror the same graduations between the groups. However, the analysis of intervallic treatment patterns (d1-d3) highlighted, independent of the different treatment procedures, the high re-growth rate on each specimen after 24 h. Considering only the metabolic activity of the different groups after 24 h (baseline of d2 or d3, Table 1), there seem no differences at all. Biofilm reduction of over 63 - 83 % using an oral irrigator results in same biofilm activity as in the control group (CO) with no treatment, after 24 h. However, this study mainly investigated metabolic activity of biofilms. Differences in pathogenicity of treated or untreated biofilms were not analyzed. Also, results of intervallic treatment were only shown over a period of three days. Biofilm regrowth and resistance to treatment may change after longer cleaning periods.

The application time of the oral irrigator was set at 10 s. Still, residual activity in a range of 17–37 % was measured. Regarding the SEM images with the oral irrigator, shorn-off matrix regions and residual biofilm areas in the outer disc regions are shown. This may be explained by the central water-jet of the oral irrigator, which seems to be very defined and does not strike the whole surface of the biofilm-coated discs to the same extent. Marginal regions of the disc, which were not in direct contact with the water-jet may result in higher amounts of residual bacteria due to less shear force. Also, the use of batch biofilms leads to bacterial adherence on all disc surfaces. The sides of the discs were not reached by oral devices and might harbor residual bacteria. The oral irrigator was applied perpendicular to the interproximal space, leading to a tangential jet to the biofilm surface. Single bacteria on the sides of the specimens may have remained untreated, however, their influence seems rather negligible due to their minor amount. Biofilm activities of 59 – 85 % remained after non-contact cleaning with the sonic toothbrush for 10 s. Previous studies have reported non-contact biofilm removal of more than 50 % by side-to-side toothbrushes [1, 2, 17–19]. Most of these studies were analyzed microscopically after staining using a confocal laser scanning microscope. Selected areas of the treated surfaces were scanned and remaining biofilm volumes were calculated and set into relation to control groups. In contrast to the methodology used in the present study, only treated surface areas were included to the analysis. However, these studies fail to compare each specimen separately prior to and after each treatment. Microscopic analysis provides only insight to the specimens after treatment, due to irreversible staining procedures. The use of alamarBlue allows repeatable measurements. Each specimen can be analyzed at different time points and effects of intervallic treatments can be observed using the identical specimen. Its application for biofilm quantification was investigated in several studies before [20, 21]. It was also validated for the three-species biofilm used in the experiments (see Additional file 1).

Since most investigations on the so called ‘non-contact biofilm removal’ were performed by directly positioned oral devices towards the center of the biofilm coated disc surfaces instead of using interproximal devices, and due to the high variety of application time, distance to specimen surfaces and biofilm models used, comparisons between single studies should be made only very carefully.

Differences between the biofilm models can also be observed with reference to the surface material used as substratum. Human enamel sections are often substituted by titanium [4], glass [1, 3, 6, 17] or hydroxyapatite discs [2, 14, 18]. The present study was performed with hydroxyapatite discs as tooth enamel analogue to avoid possible inhomogeneous bacterial adhesion patterns along enamel cracks and fissures.

With respect to the better performance of the oral irrigator as compared to the activated sonic toothbrush one has to bear in mind that in both cases no toothpaste was involved, although by using a toothbrush this is rarely the case. The additional use of toothpaste together with the activated sonic toothbrush may have lead to further removal of biofilm due to a mechanical effect of particles from the toothpaste, and also due to further ingredients e.g. tensides. Therefore, for further studies the additional influence of toothpaste should be investigated, as well. However, it was the intention of this study to investigate the specific influence of the devices without interference by other factors. For further research, the effect of longer intervallic cleaning procedures would help understanding the long-term effect of different oral hygiene pattern on interproximal biofilms. The comparison of non-contact brushing by interproximal devices and by non-contact brushing by directly positioned oral devices towards the biofilms would facilitate comparisons between different study models.

## Conclusions

Based on the results, cleaning of interproximal regions by hydrodynamic flow of an oral irrigator may achieve more effective removal of interproximal biofilm compared to sonic toothbrushes.

## Additional file

Additional file 1:
**Validation of the alamarBlue assay.** (DOCX 194 kb)

## Abbreviations

CFU: Colony forming units
CO: Control specimens (biofilm-coated specimens without treatment)
d1: First day of treatment
d2: Second day of treatment
d3: Third day of treatment
mFUM: modified fluid universal medium
NaCl: Sodium chloride
ORS: Oral irrigation systems
rfu: Relative fluorescence units
SEM: Scanning electron microscope
WF: Waterpik® Sensonic WP-100E: oral irrigator
WPa: Waterpik® Sensonic SR-3000E: sonic toothbrush, active
WPi: Waterpik® Sensonic SR-3000E: sonic toothbrush, inactive
