# Supplementary material for: Interproximal biofilm removal by intervallic use of a sonic toothbrush compared to an oral irrigation system
Source: BMC Oral Health. 2015 Aug 5;15:91. doi: 10.1186/s12903-015-0079-6 (PMC4526281; doi:10.1186/s12903-015-0079-6)
Supplement: Additional file 1: — Validation of the alamarBlue assay. (DOCX 194 kb) [file 12903_2015_79_MOESM1_ESM.docx]

Additional file 1 Validation of the alamarBlue assay

left: Different dilution series (n = 10 per dilution) of the inoculum (*S. mutans*, *S. oralis*, *A. naeslundii*) were plated out (CFU) and measured using the alamarBlue assay over time until complete substrate conversion (resazurin reduction to resorufin). Fluorometrical measurements were conducted every 15 min for 2 h. The measured relative fluorescence units (rfu) of each dilution were plotted against the incubation time. The dashed lines illustrate linear substrate conversion rate of alamarBlue (initial 30% of total conversion). All measurements of the experiment were conducted within this range.

right: The inter-assay variation (recovery and coefficient of variation (COV) of 10 repeats) illustrates the limits of this method after 30 min. Recovery for CFU resulted in 94-102%. The COV of 20% limited the lower level of quantification to 50 x 10^6^ CFU/ml.
